# Excellent Pathologic Response and Atypical Clinical Course of High-Grade Extremity Sarcoma to Neoadjuvant Pencil Beam Scanning Proton Therapy

**DOI:** 10.7759/cureus.1687

**Published:** 2017-09-15

**Authors:** Jill Remick, William Regine, Robert Malyapa, Vincent Ng, Melissa Vyfhuis, Tejan Diwanji, Susan Shyu, James W Snider

**Affiliations:** 1 Radiation Oncology, University of Maryland School of Medicine; 2 Department of Orthopaedics, University of Maryland School of Medicine; 3 Department of Pathology, University of Maryland Medical Center

**Keywords:** soft tissue sarcoma, relative biological effectiveness, image guidance, igrt, high grade sarcoma, proton therapy, pencil beam scanning proton therapy, sarcoma, complete response, pbs

## Abstract

Neoadjuvant radiation therapy, followed by definitive surgical resection, remains the standard of care for resectable high-grade and unresectable soft tissue sarcomas. Proton therapy offers the promise of highly conformal dose distributions with improved sparing of neighboring normal tissues as compared with conformal and intensity modulated photon techniques. It is unclear whether proton therapy may offer an improved tumoral response, especially with dose escalation, in this relatively radio-insensitive tumor type. We, herein, present a patient with an excellent pathologic response to preoperative pencil beam scanning proton therapy despite a complex treatment course.

## Introduction

Accounting for approximately 1% of all adult tumors, soft tissue sarcoma (STS) remains a rare disease entity with over half arising in the extremity [1]. The combination of radiotherapy (RT) with limb-sparing surgery, particularly in patients with high-grade disease, has proven equally efficacious to amputation and remains the standard of care [2]. Preoperative RT is now generally preferred owing to its inherent reductions in RT-treated volumes and dose, which have been linked to long-term complications and worsened limb function [3]. However, traditional two dimensional (2D) and three dimensional (3D) conformal RT (3D-CRT) techniques have historically produced relatively high rates of major wound complications (> 30%) with this preoperative approach. 

More conformal techniques, e.g. intensity modulated radiation therapy (IMRT) and reduced treatment volumes have each effected lower rates of complication without sacrifices in disease control in institutional series and a major cooperative group trial, respectively [4-5]. Due to its physical properties and finite range, proton therapy (PT) offers further promise in reducing side effects from RT as well as the potential for dose escalation. However, PT does require increased clinician diligence and vigilance throughout the course of therapy as it is substantially more sensitive to density, alignment, and anatomical changes along the beam path.

Small series and dosimetric studies have suggested the utility of PT in this setting [6-7]. However, there remains some lack of clarity on the relative biological effectiveness (RBE) of PT across the spread-out Bragg peak (SOBP), especially at the end of the range of the beam, as employed in both passive scattering and pencil beam scanning (PBS) techniques [8]. A number of excellent tumoral responses and normal tissue complications have been attributed to this uncertainty in the literature. Here, we present a case of a patient with a high-grade extremity STS who underwent preoperative PBS-PT at our institution with a challenging clinical course, but an impressive tumoral response.

## Case presentation

**Figure 1:**
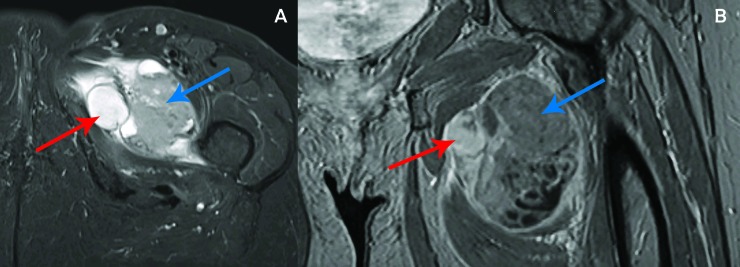
Pre-treatment magnetic resonance (MR) imaging. Axial T1 turbo inversion recovery magnitude (TIRM) (A) and coronal T1 volumetric interpolated breath-hold examination (VIBE) (B) MRI of the left upper thigh mass measuring  6.4 cm anteroposterior x 7.5 cm transverse x 7.1 cm craniocaudal shown in the axial (A) and coronal (B) plane. Note the small regions of cystic necrosis (red arrow) and surrounding soft tissue component (blue arrow).

A 62-year-old female with no major medical history initially presented with a one-month history of left hip pain and left leg swelling. Her pain progressed, and she developed trouble ambulating. This prompted diagnostic imaging, including magnetic resonance imaging (MRI) with contrast of the left femur. This study revealed a heterogeneously enhancing mass measuring 6.4 cm anterior-posterior x 7.5 cm transverse x 7.1 cm craniocaudal centered in the left abductor brevis muscle containing some cystic areas and a small amount of surrounding edema (Figure 1).  Also noted were seven non-pathologically enlarged (< 1 cm) left inguinal lymph nodes. 

A computed tomography (CT)-guided biopsy of the mass was performed, which confirmed a diagnosis of high grade undifferentiated pleomorphic sarcoma. Two lymph nodes in the groin were biopsied and were negative for metastatic involvement. Positron emission tomography-computed tomography (PET-CT) examination demonstrated a metabolically active left inner thigh mass without evidence of regional lymphadenopathy or distant metastasis. Therefore, the patient was clinically diagnosed as Stage III, T2bN0M0 (Grade 3).

She was recommended for and elected to undergo neoadjuvant radiation therapy prior to surgical resection. Given the deep-seated location of the tumor just inferior to the femoral head and neck and abutting the femoral shaft, PBS-PT was recommended to limit the dose to the left hip as well as to the vaginal structures. Dose reductions to the left thigh, femur, and hip were expected to reduce her risk of post-treatment edema or fracture.

For radiation planning, an MRI with contrast was obtained in the treatment position and fused with the planning CT. As per our institutional guidelines and in concordance with the Radiation Therapy Oncology Group (RTOG) Phase II Protocol 0630 for Neoadjuvant Radiation for Soft Tissue Sarcoma, the gross tumor volume (GTV) was contoured based on post-gadolinium T1 imaging and expanded by 3 cm superiorly and inferiorly and 1.5 cm radially, respecting normal tissue and compartmental boundaries (unless apparently involved), to generate the clinical target volume (CTV).  All T2 flair changes were included in the CTV, if not covered by the expansion. A planning target volume (pPTV) was generated based on lateral setup uncertainty of 5 mm and beam-specific range uncertainty. However, robust evaluation of CTV coverage was assessed for plan approval. For image guidance during treatment, kilovoltage (kV) images were performed daily and aligned to the bone. Quality assurance computed tomography (QA-CT) scans were performed at week one and week three of treatment to evaluate for soft tissue swelling and tumor changes. An additional QA-CT was performed in the latter half of her treatment due to increased swelling noted on exam.

**Figure 2:**
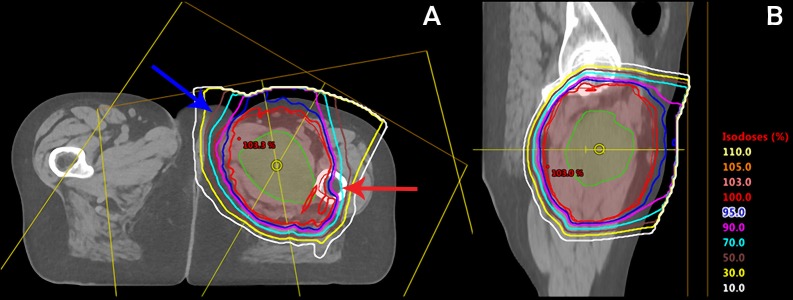
Neoadjuvant pencil beam scanning proton therapy (PBS-PT) plan Proton radiation plan which includes two co-planar beams entering anteriorly with gantry angles of 30 degrees and 350 degrees (A) and sagittal view (B). The planning target volume (PTV) is depicted in red colorwash. Note the relative sparing of the femoral shaft (red arrow) and the genitals/labia (blue arrow).

**Figure 3:**
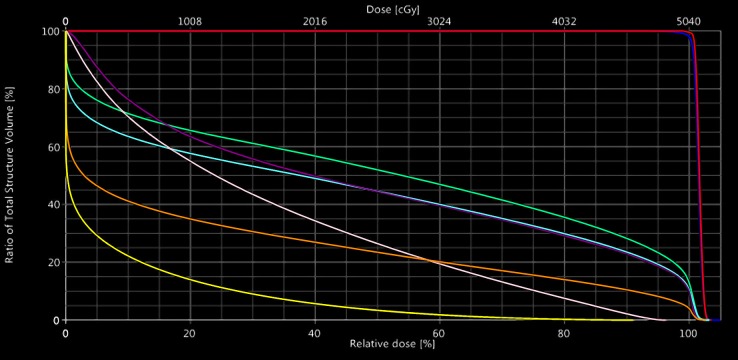
Pencil beam scanning proton therapy dose volume histogram GTV (red), CTV (dark blue), left femoral head (green), left hip (light blue), joint space (purple), labia (pink), acetabulum (orange), and bladder (yellow). GTV: gross tumor volume; CTV: clinical target volume

The dose prescribed was 50.4 Gy RBE in 1.8 Gy RBE per fraction. The proton plan employed a single field optimization (SFO) technique with two coplanar fields entering anteriorly at gantry angles of 30 degrees and 350 degrees, respectively (Figure 2). The left femoral head received a mean dose of 24.4 Gy RBE, which met the constraint of the mean dose < 37 Gy RBE. The mean dose to the left hip joint (which included the left femoral head, joint space, and acetabulum) was 21.1 Gy RBE (Figure 3). 

**Figure 4:**
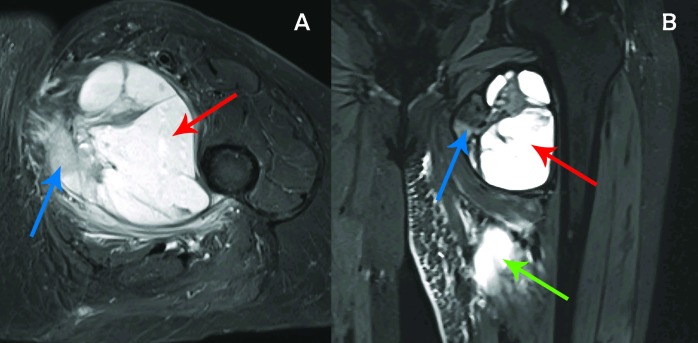
Mid-treatment magnetic resonance imaging Mid-treatment MRI (T1 TIRM) revealing interval growth in the mass now measuring 8.2 cm x 8.5 cm x 9.5 cm in the axial (A) and coronal (B) views. Note the region of large cystic necrosis (red arrow) and soft tissue encapsulated component (blue arrow). Also, note the increased muscular edema (green arrow) which extends inferiorly to below midshaft of femur. MRI: magnetic resonance imaging; TIRM: turbo inversion recovery magnitude

The patient developed significant swelling and pain during the course of treatment. She also experienced intermittent fevers and sweats. Infectious workup, including complete blood count (CBC), urinalysis, and chest x-ray, was repeatedly negative. Her pain worsened significantly over a short period of time, which completely impaired her ability bear weight on the affected leg. She underwent a repeat MRI midway through treatment that revealed the mass to appear noticeably larger, measuring 8.2 x 8.5 x 9.5 cm, with a large cystic region within the mass and increased surrounding muscular edema (Figure 4). Medially, there was a soft tissue encapsulated component which measured 4.8 cm. Overall, these findings were concerning for possible tumor progression versus treatment reaction. After 23 fractions (41.4 Gy RBE), the PBS-PT plan was re-planned due to observed tumor growth on exam and imaging. The prescription dose for the re-plan was 9 Gy RBE for a total dose of 50.4 Gy RBE. The mean relative dose (given the different prescription doses for the initial and re-plan) to the left hip and femoral head was higher with the re-plan when compared to the initial proton plan (85% vs 42% and 93% vs 48%, respectively) as we expected, given the significant increase in size of the target volume. 

By the end of treatment, the patient was generally wheelchair-bound; however, she could bear some weight on her left leg.

**Figure 5:**
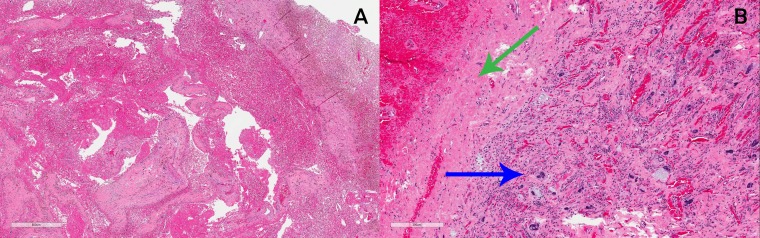
Final pathology from resected specimen Post-radiation tissue pathology of left thigh resection, hematoxylin and eosin stain. (A) Low power magnification: soft tissue mass with extensive hemorrhagic and fibrinopurulent necrosis. (B) High power field: small focus of residual viable tumor (blue arrow) composed of highly pleomorphic cells with marked atypia and inflamed collagenous stroma in a background of extensive hemorrhagic necrosis (green arrow).

Due to her symptomology and concern for progression, the patient was taken to the operating room only 12 days after completion of radiation therapy. A gross total resection with primary closure was achieved without complication. Final pathology revealed an undifferentiated pleomorphic sarcoma, measuring 9.5 cm with negative resection margins. The closest margin was 3 mm at the deep margin but represented the periosteal layer. A near complete response was noted with greater than 80% of the tumor necrotic, consistent in appearance with treatment effect (Figure 5). The cells demonstrated rare positivity for desmin and negativity for pan cytokeratin and S100. Pathologically, she was staged at ypT2bN0Mx.

**Figure 6:**
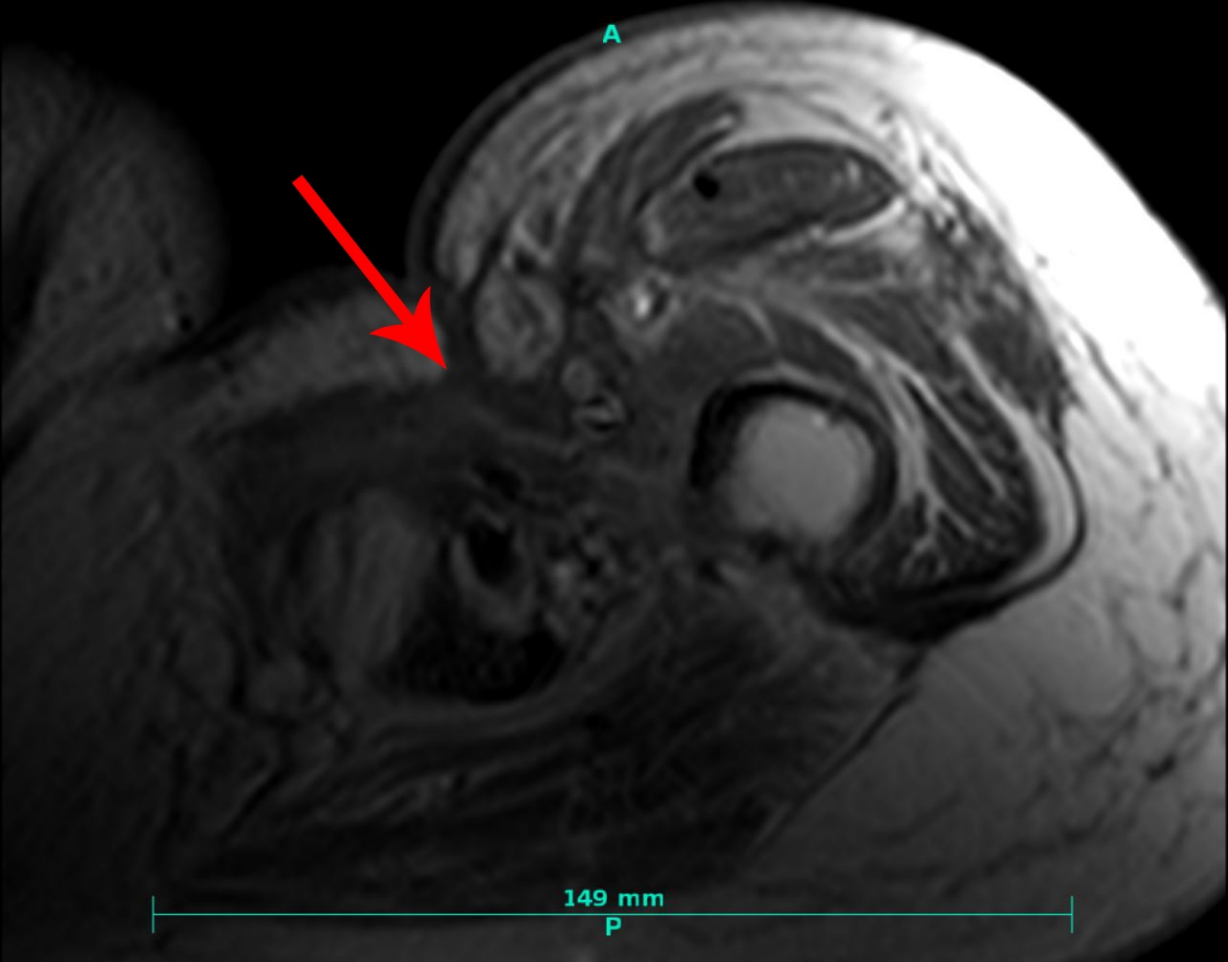
Four month follow-up magnetic resonance examination Note in this axial T1 post-contrast image the postoperative change and scar tissue formation in the surgical bed (red arrow) with no evidence of mass or recurrence at this juncture four months post-treatment.

The patient, unfortunately, developed a postoperative wound complication with associated infection. She was readmitted approximately two weeks after surgery for intravenous (IV) antibiotics and surgical debridement with the application of a vacuum-assisted closure ((VAC) device. One month following this procedure, her wound was noted to be healing nicely. MRI performed four months post-treatment demonstrated no evidence of recurrent disease (Figure 6).

## Discussion

PBS-PT offers substantial dosimetric benefits over 3D-CRT and IMRT in the treatment of extremity STS that could lead to meaningful improvements in clinical outcomes, especially with regard to acute wound/bone and long-term limb toxicity [6]. Beyond the target, PT reduces exposure of normal tissue compartments and bone to moderate doses of RT that contribute to wound complications, fibrosis, lymphedema, and bone fractures. Proximally, PT has generally been considered to be non-skin sparing due to its relatively high entrance dose in targets close to the skin surface; yet, with intensity modulation (IMPT) and the use of multiple beam angles, this effect can be somewhat mitigated.

To date, there is relatively limited data regarding outcomes following PT for STS, especially in the extremity subset. The University of Pennsylvania has recently presented results with PT for sarcomas arising in previously irradiated fields in 23 patients [7]. High-grade toxicity was limited with only a 15% rate of significant wound complications. Of the 10 extremity patients included, seven were spared amputation (70%). Additional results are eagerly awaited in both the extremity and retroperitoneal disease entities with several Phase I and II trials currently underway.

The current case presented, however, offers a number of critical clinical points to consider. First, a substantial increase in patient discomfort and tumoral volume must always raise concern for tumoral progression under treatment. This necessitates consideration of early surgical intervention or cessation of the neoadjuvant approach. However, tumoral swelling is also a well-documented phenomenon that should give some pause before aborting the planned course of therapy. In this case, a reasonable balance was struck by completing the preoperative RT expediently but then proceeding to surgical resection earlier than intended (12 days instead of four to six weeks).

The patient did require aggressive wound care and antibiotics, consistent with wound complications occurring in approximately one-third of patients receiving neoadjuvant RT for STS [3]. The near doubling of the risk of wound complications encountered with neoadjuvant therapy (as opposed to postoperative RT) is considered acceptable to reduce treatment volumes and dose, which has been correlated with longer-term morbidities. In this case, such a complication was not surprising based on the truncated timeline to resection. A preoperative healing period of four to six weeks likely would have reduced the risk of complication, but due to patient symptomology, it was not feasible in this case.

The critical importance of image-guidance and clinical vigilance in the delivery of PT is also highlighted. A tumoral volume change such as this may not compromise a 3D-CRT plan with adequate field "flash" or even an IMRT plan with numerous beam angles, but PT is highly sensitive to such anatomical changes. This swelling, if unmonitored, could have led to dramatic dose changes in tumor coverage and organs-at-risk (OAR) exposure. Image guidance with intermittent 3D imaging is a must. Re-simulation with an assessment of dose distribution (with re-planning as necessary) should be standardly employed when anatomical changes present during the delivery of PT, as was done in this case. Beam angles and delivery techniques, e.g. single field optimization (SFO) that increase robustness to such changes, should be consciously sought in the PT planning process if acceptable tumor coverage and sparing of OARs can be achieved.

The impressive pathologic tumoral response is very encouraging. Pathologic response after neoadjuvant photon radiation for STS has been previously reported in a retrospective setting to range from 5% - 100% with a median response rate of 35% [9]. In this series, 27% of tumors demonstrated greater than 80% necrosis after neoadjuvant radiation therapy and 10% showed a complete pathologic response (defined as > 95% necrosis). They further reported that disease-free survival at three years was numerically improved after a pathologic complete response, 100% compared to 63% in incomplete responses, although this difference was not statistically significant.

Another study found a median pathologic response of 67.5% for low-grade and 50% for high-grade tumors [10]. In the current case, our patient experienced an impressive pathologic response (> 80%) despite the large and high-grade nature of the tumor.

The exact mechanism for the rapid necrosis and cystic degeneration of this patient’s tumor is unclear. However, PT treatment effect from direct cellular damage with or without resultant vascular compromise is suspected. Considering the excellent pathologic response with minimal residual disease at only 12 days post-PT, it is likely that, given a normal post-neoadjuvant interval of several weeks, a pathologic complete response may have been achieved at resection. It is unclear whether PT will offer improved tumoral responses over traditional photon techniques in this relatively radio-insensitive histology or similar tumor types. Improved modeling of RBE across the PT beam is eagerly sought and may clarify these effects. However, PT’s potential for dose-escalation without substantially added toxicity holds promise for an improved therapeutic ratio. Additional research is justified to clarify the role of PT in STS.

## Conclusions

PBS-PT is safe and effective for the delivery of neoadjuvant RT prior to definitive surgical resection for high-grade or unresectable STS. PT may offer substantial sparing of normal tissues, as well as increased RBE across the SOBP (requiring even closer clinical monitoring), over that achievable with conformal photon techniques. An excellent tumoral pathologic response was achieved in this case, though it was clinically confounded by tumor volume increase and worsened patient discomfort. Additional investigation is required to optimize proton therapy delivery and RBE characterization.

## References

[1] Hoefkens F, Dehandschutter C, Somville J (2016). Soft tissue sarcoma of the extremities: pending questions on surgery and radiotherapy. Radiat Oncol.

[2] Rosenberg SA, Tepper J, Glatstein E (1982). The treatment of soft-tissue sarcomas of the extremities: prospective randomized evaluations of (1) limb-sparing surgery plus radiation therapy compared with amputation and (2) the role of adjuvant chemotherapy. Ann Surg.

[3] O'Sullivan B, Davis AM, Turcotte R (2002). Preoperative versus postoperative radiotherapy in soft-tissue sarcoma of the limbs: a randomised trial. Lancet.

[4] Folkert MR, Singer S, Brennan MF (2014). Comparison of local recurrence with conventional and intensity-modulated radiation therapy for primary soft-tissue sarcomas of the extremity. J Clin Oncol.

[5] Wang D, Zhang Q, Eisenberg BL (2015). Significant reduction of late toxicities in patients with extremity sarcoma treated with image-guided radiation therapy to a reduced target volume: Results of Radiation Therapy Oncology Group RTOG-0630 Trial. J Clin Oncol.

[6] Fogliata A, Scorsetti M, Navarria P (2013). Dosimetric comparison between VMAT with different dose calculation algorithms and protons for soft-tissue sarcoma radiotherapy. Acta Oncol.

[7] Guttmann DM, Frick MA, Carmona R (2017). A prospective study of proton reirradiation for recurrent and secondary soft tissue sarcoma. Radiother Oncol.

[8] Guan F, Bronk L, Titt U (2015). Spatial mapping of the biologic effectiveness of scanned particle beams: towards biologically optimized particle therapy. Sci Rep.

[9] Shah D, Borys D, Martinez SR (2012). Complete pathologic response to neoadjuvant radiotherapy is predictive of oncologic outcome in patients with soft tissue sarcoma. Anticancer Res.

[10] Roberge D, Skamene T, Nahal A (2010). Radiological and pathological response following pre-operative radiotherapy for soft-tissue sarcoma. Radiother Oncol.

